# Poached Egg Lesions in the Colon Because of Amebiasis

**DOI:** 10.4269/ajtmh.24-0852

**Published:** 2025-04-22

**Authors:** Lalit Chandra Kummetha, Venkatesh Vaithiyam, Surbhi Goyal

**Affiliations:** ^1^ Department of Gastroenterology, Govind Ballabh Pant Hospital and Associated Maulana Azad Medical College, New Delhi, India;; ^2^ Department of Pathology, Govind Ballabh Pant Hospital and Associated Maulana Azad Medical College, New Delhi, India

**Figure 1.**
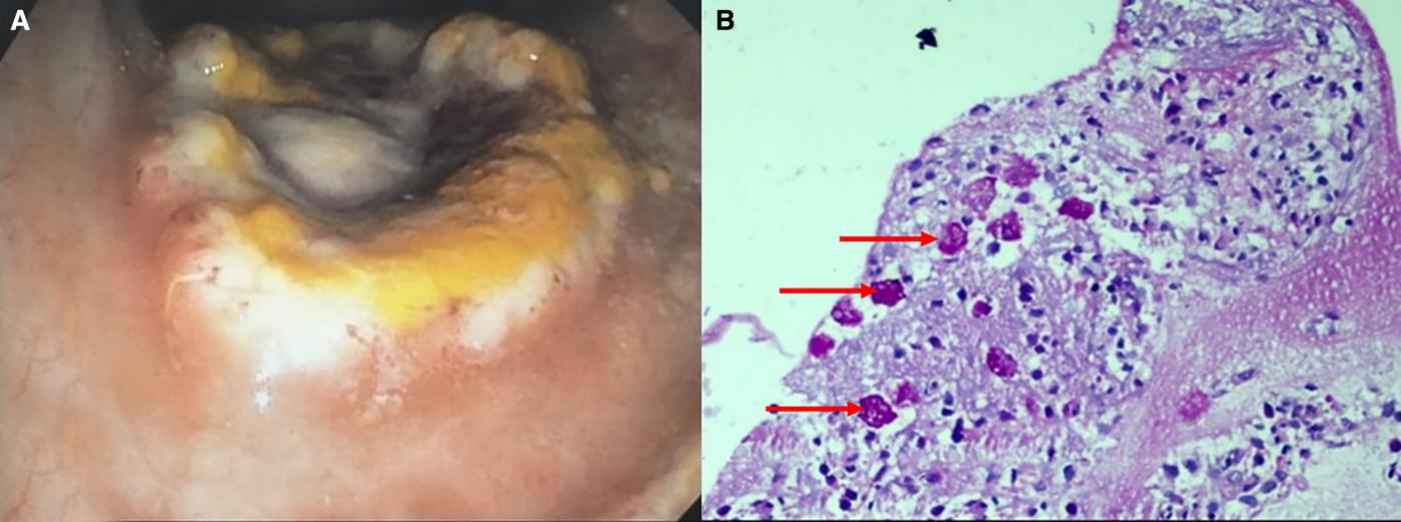
(**A**) Colonoscopic image shows a large irregular ulcer with yellowish necrotic material on top of whitish slough giving a “poached egg appearance.” (**B**) Histopathological examination from the colonic ulcer shows numerous trophozoites of *Entamoeba histolytica* highlighted by Periodic Acid Schiff (Red arrows) (H&E ×400).

A 75-year-old woman presented with abdominal pain, diarrhea, urgency, and tenesmus for 3 weeks. She also complained of blood-mixed stools for the initial 3 days. Examination and blood investigations were normal except for hypoalbuminemia and positive amoebic serology. The stool examination showed no ova, cysts, trophozoites, or atypical organisms. In view of the persistent symptoms, a colonoscopy was performed, which revealed multiple ulcers with yellowish necrotic material on the top of the whitish slough, giving a “poached egg appearance” in the ascending colon and rectum (Figure 1A). Histopathological examination of the ulcers showed numerous trophozoites of *Entamoeba histolytica* (Figure 1B). The patient was treated with metronidazole, and she recovered completely.

Colonic amoebiasis is a significant health burden, particularly in low-income countries. Noninvasive investigations like stool microscopy have low sensitivity and specificity, whereas serological tests to detect IgG antibodies to trophozoites have low sensitivity and are not useful in endemic regions. Stool antigen test detecting Gal/GalNAc lectin has higher reported sensitivity and specificity, approaching 80%.^1^ DNA amplification tests such as stool polymerase chain reaction are now available and target specific genes with up to 100% sensitivity and specificity.^1^ However, these tests are limited by cost constraints in low-income countries.

Colonoscopy is indicated for patients with persistent gastrointestinal symptoms. The most common sites of involvement are the cecum and ascending colon, where excystation and trophozoite release occur. Ulcers range from small to large (>2 cm) and are usually multiple and discrete.^2^ As shown in this case, a poached egg appearance has been described and is likely an uncommon manifestation, but characteristic of intestinal amoebiasis.^3^
